# Supplementary material for: The Childbirth Fear Questionnaire and the Wijma Delivery Expectancy Questionnaire as Screening Tools for Specific Phobia, Fear of Childbirth
Source: Int J Environ Res Public Health. 2022 Apr 12;19(8):4647. doi: 10.3390/ijerph19084647 (PMC9028446; doi:10.3390/ijerph19084647)
Supplement: Supplementary file 1 [file ijerph-19-04647-s001.zip › ijerph-1591879-supplementary.pdf]

Supplementary material

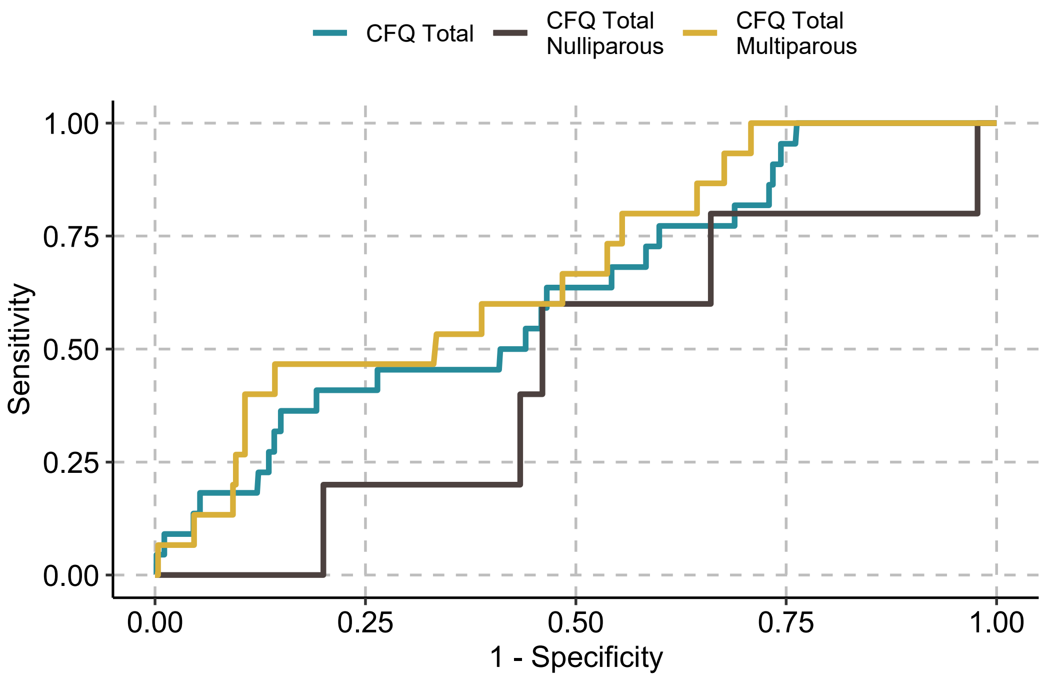

Figure S1. Receiver operating characteristic (ROC) curves for the Childbrith Fear Questionnaire (CFQ) across parity (full diagnostic criteria ONLY).

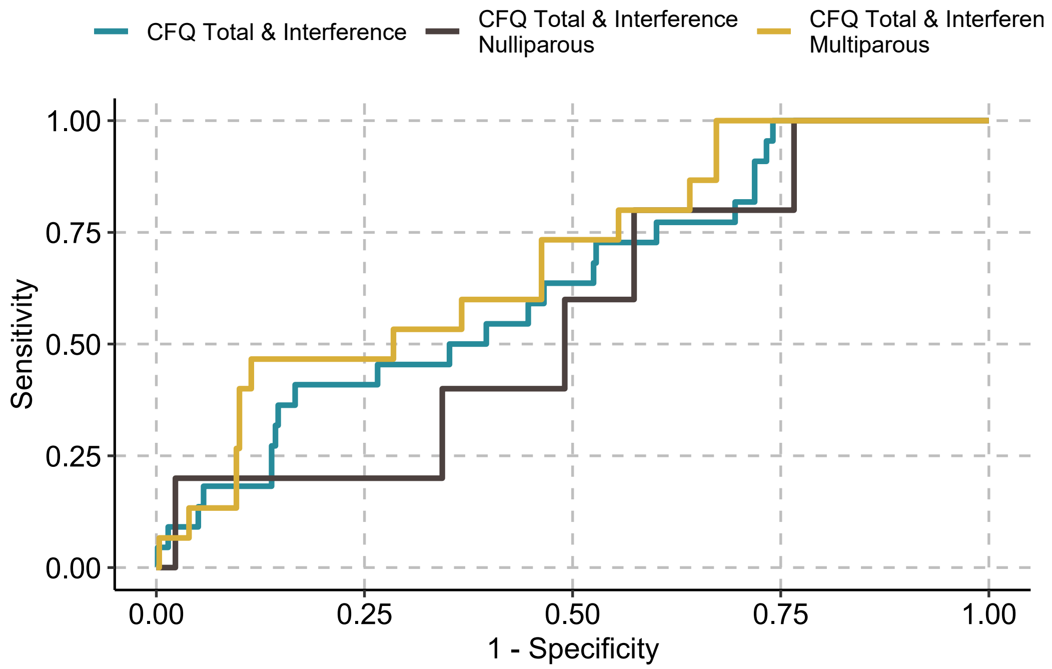

Figure S2. ROC curves for the CFQ (Total and Interference Subscale scores) across parity (full diagnostic criteria ONLY).

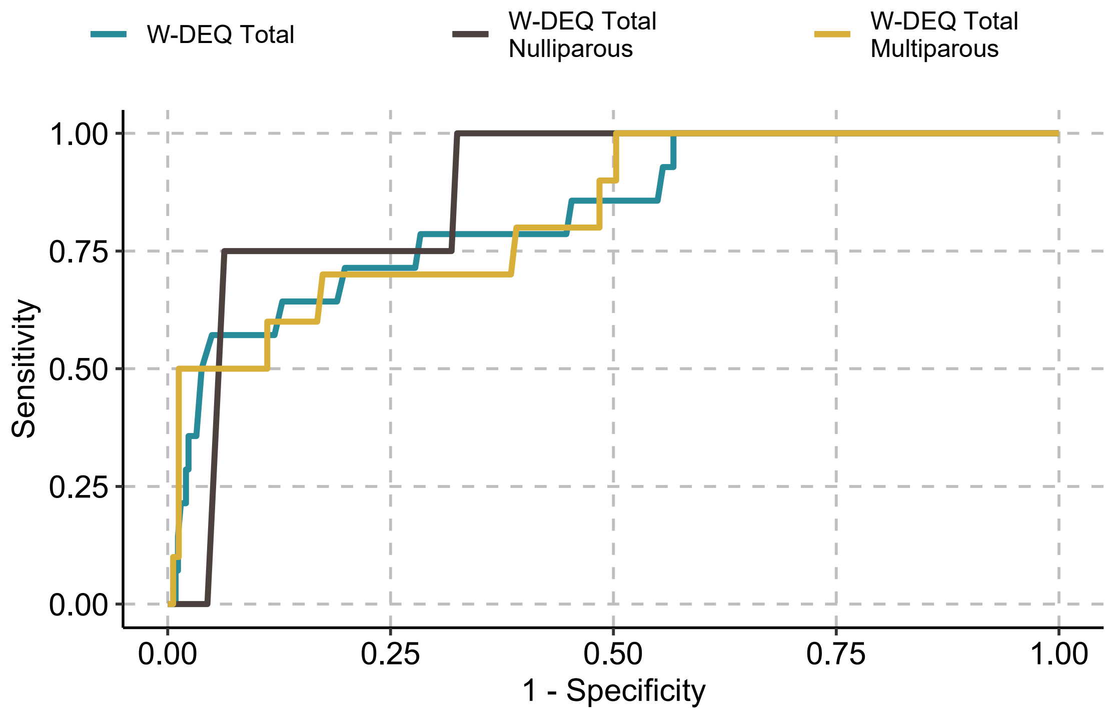

Figure S3. ROC curves for the Wijma Delivery Expectations Questionnaire (W-DEQ) across parity (full diagnostic criteria ONLY).

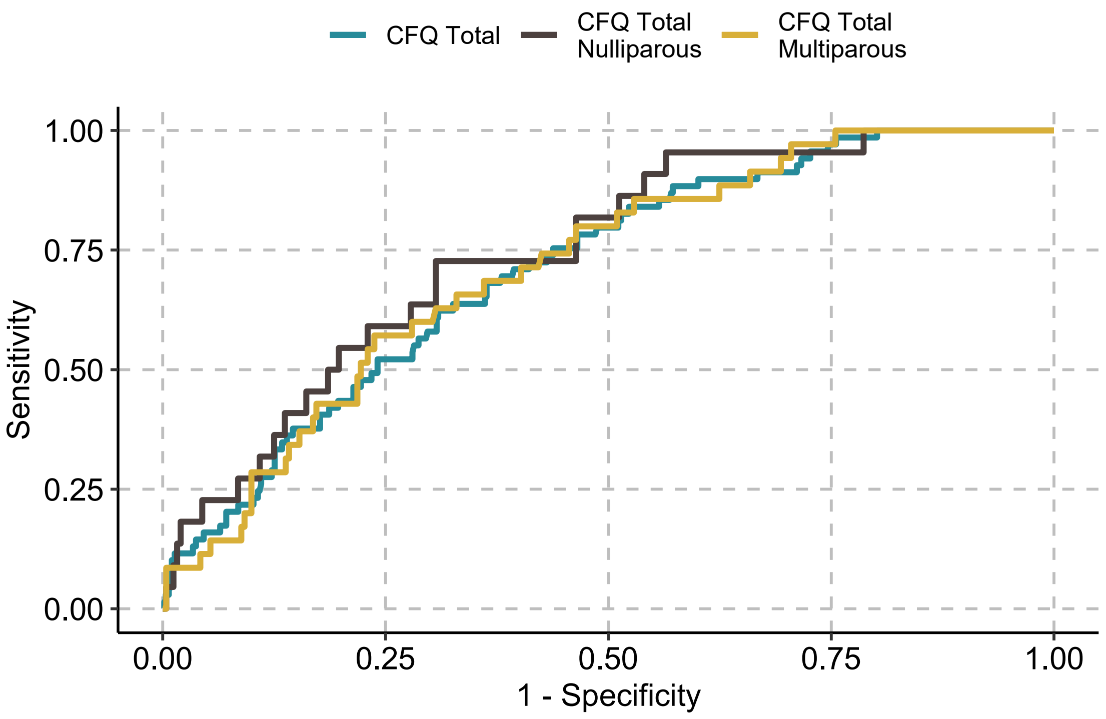

Figure S4. ROC curves for the CFQ across parity (subclinical and full diagnostic criteria combined).

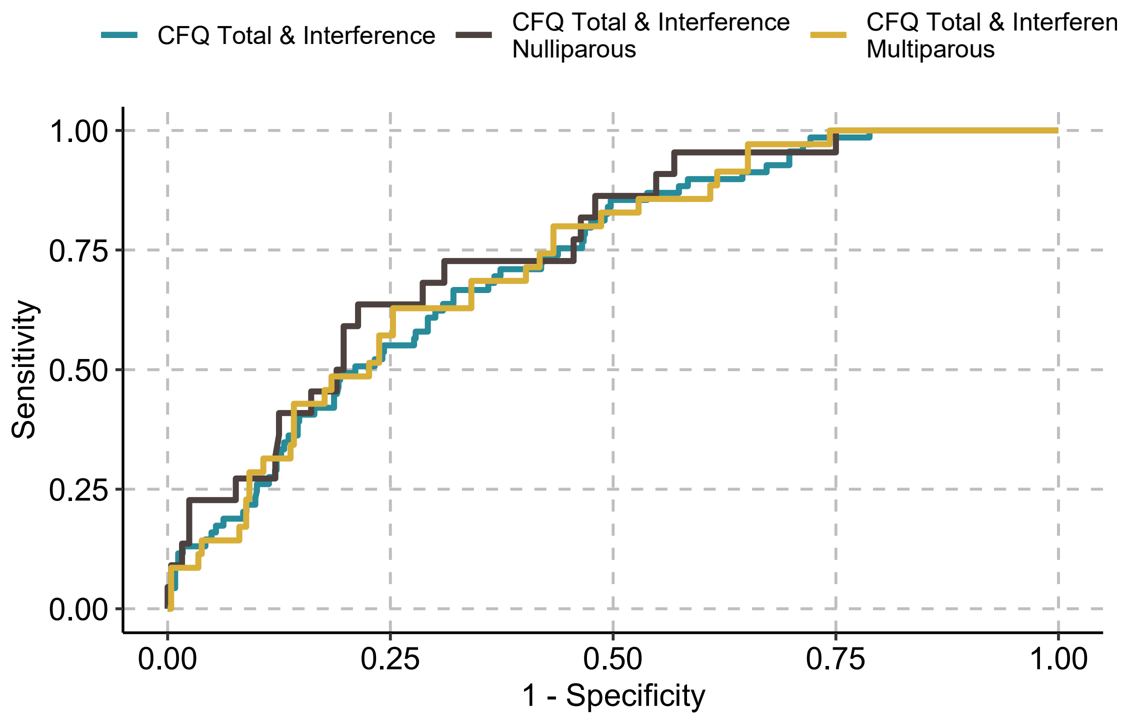

Figure S5. ROC curves for the CFQ (Total and Interference Subscale scores) across parity (subclinical and full diagnostic criteria combined).

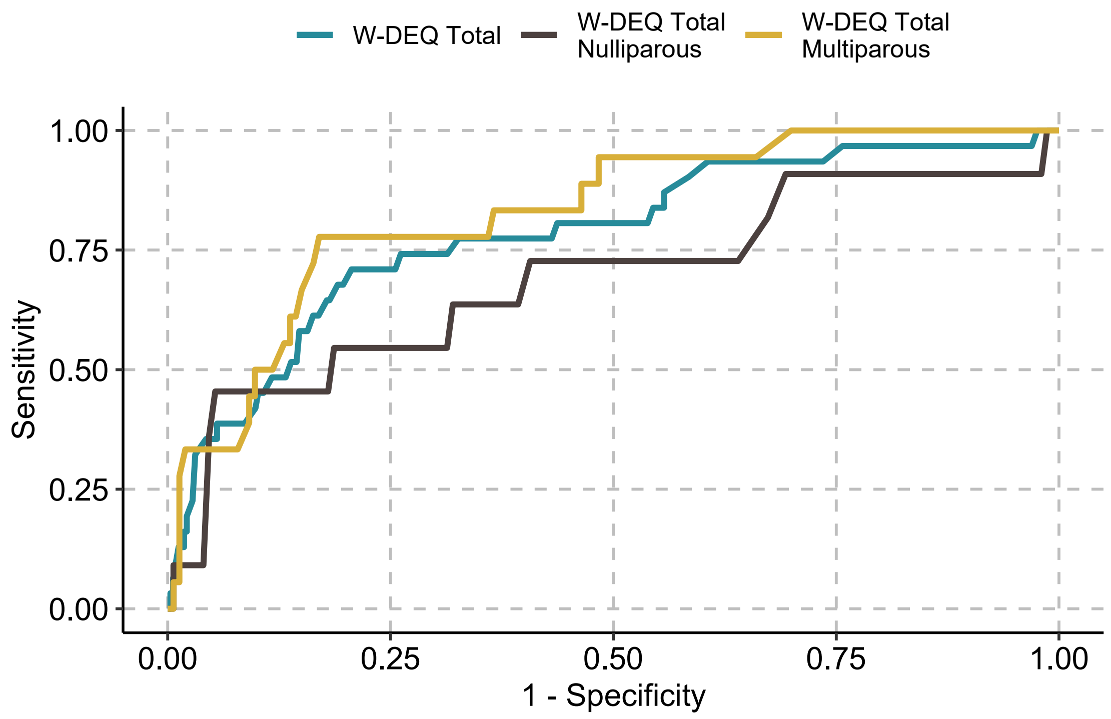

Figure S6. ROC curves for the W-DEQ across parity (subclinical and full diagnostic criteria combined).

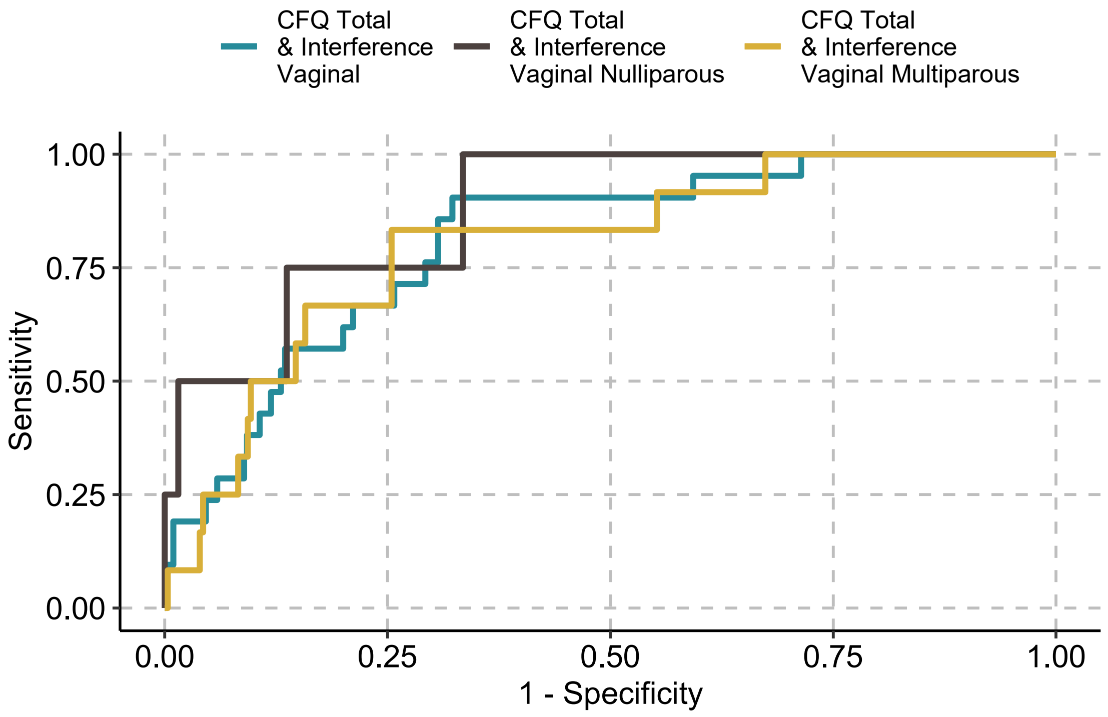

Figure S7. ROC curves for the CFQ across parity, separately for fear of vaginal birth (subclinical and full diagnostic criteria combined)

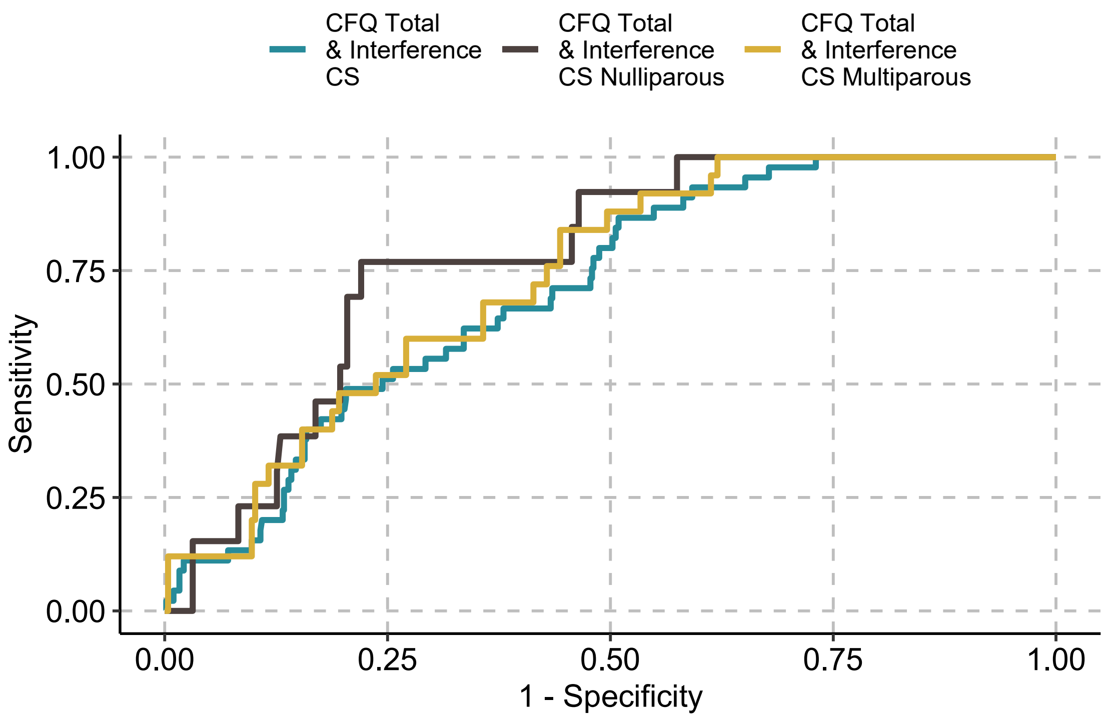

Figure S8. ROC curves for the CFQ across parity (Total and Interference Subscale scores), separately for fear of cesarean birth (CS; subclinical and full diagnostic criteria combined).

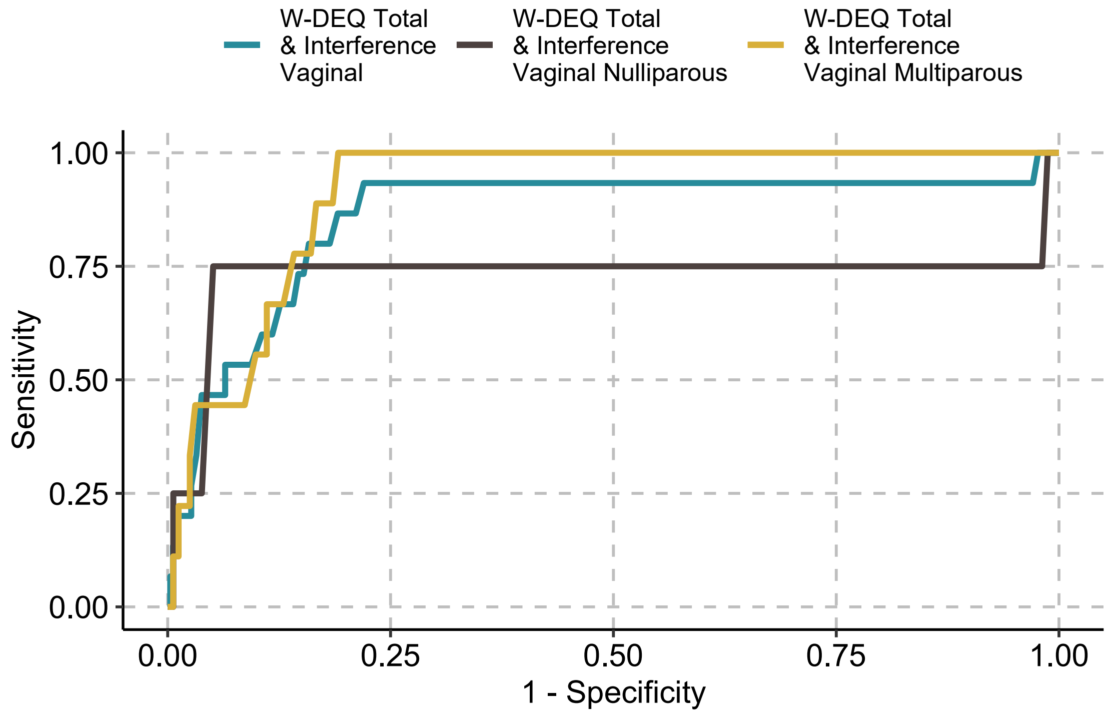

Figure S9. ROC curves for the W-DEQ across parity, separately for fear of vaginal birth (subclinical and full diagnostic criteria combined)
